# Modeling Risk Factors for Sleep- and Adiposity-Related Cardiometabolic Disease: Protocol for the Short Sleep Undermines Cardiometabolic Health (SLUMBRx) Observational Study

**DOI:** 10.2196/27139

**Published:** 2021-03-09

**Authors:** Adam P Knowlden, John C Higginbotham, Michael A Grandner, John P Allegrante

**Affiliations:** 1 Department of Health Science College of Human Environmental Sciences The University of Alabama Tuscaloosa, AL United States; 2 Department of Community Medicine and Population Health College of Community Health Sciences The University of Alabama Tuscaloosa, AL United States; 3 Department of Psychiatry College of Medicine University of Arizona Tucson, AZ United States; 4 Department of Health and Behavior Studies Teachers College Columbia University New York, NY United States; 5 Department of Sociomedical Sciences Mailman School of Public Health Columbia University New York, NY United States

**Keywords:** abdominal obesity-metabolic syndrome, adiposity, body composition, body fat distribution, insufficient sleep syndrome, observational study, short sleeper syndrome, sleep deprivation

## Abstract

**Background:**

Obesity and short sleep duration are significant public health issues. Current evidence suggests that these conditions are associated with cardiovascular disease, metabolic syndrome, inflammation, and premature mortality. Increased interest in the potential link between obesity and short sleep duration, and its health consequences, has been driven by the apparent parallel increase in the prevalence of both conditions in recent decades, their overlapping association with cardiometabolic outcomes, and the potential causal connection between the two health issues. The SLUMBRx (Short Sleep Undermines Cardiometabolic Health) study seeks to contribute to the development of a comprehensive adiposity-sleep model while laying the groundwork for a future research program that will be designed to prevent and treat adiposity- and sleep-related cardiometabolic disease risk factors.

**Objective:**

This SLUMBRx study aims to address 4 topics pertinent to the adiposity-sleep hypothesis: the relationship between adiposity and sleep duration; sex-based differences in the relationship between adiposity and sleep duration; the influence of adiposity indices and sleep duration on cardiometabolic outcomes; and the role of socioecological factors as effect modifiers in the relationship between adiposity indices, sleep, and cardiometabolic outcomes.

**Methods:**

SLUMBRx will employ a large-scale survey (n=1000), recruiting 159 participants (53 normal weight, 53 overweight, and 53 obese) to be assessed in 2 phases.

**Results:**

SLUMBRx was funded by the National Institutes of Health, Heart, Lung, and Blood Institute through a K01 grant award mechanism (1K01HL145128-01A1) on July 23, 2019. Institutional Review Board (IRB) approval for the research project was sought and obtained on July 10, 2019. Phase 1 of SLUMBRx, the laboratory-based component of the study, will gather objective adiposity indices (air displacement plethysmography and anthropometrics) and cardiometabolic data (blood pressure, pulse wave velocity and pulse wave analysis, and a blood-based biomarker). Phase 2 of SLUMBRx, a 1-week, home-based component of the study, will gather sleep-related data (home sleep testing or sleep apnea, actigraphy, and sleep diaries). During phase 2, detailed demographic and socioecological data will be collected to contextualize hypothesized adiposity and sleep-associated cardiometabolic disease risk factors. Collection and analyses of these data will yield information necessary to customize future observational and intervention research.

**Conclusions:**

Precise implementation of the SLUMBRx protocol promises to provide objective and empirical data on the interaction between body composition and sleep duration. The hypotheses that will be tested by SLUMBRx are important for understanding the pathogenesis of cardiometabolic disease and for developing future public health interventions to prevent its conception and treat its consequences.

**International Registered Report Identifier (IRRID):**

PRR1-10.2196/27139

## Introduction

### Background

Obesity and short sleep in adults are highly prevalent in the United States [1]. Both conditions are associated with numerous adverse health outcomes, including all-cause mortality [2], cardiovascular disease [3], diabetes [4], metabolic syndrome [5], inflammation [6], and psychiatric disorders [7]. Approximately 38% of adults in the United States are classified as obese, with a BMI of 30 kg/m^2^ or greater [8]. Stratified by sex, 35.2% of men and 40.5% of women meet the BMI cutoff point for obesity [8]. Similarly, short sleep is common, with an estimated 35.3% of adults in the United States receiving less than the recommended 7 hours of sleep during a 24-hour period [9]. Among this sample, 56.5% of men and 39.6% of women self-reported snoring [9], a symptom associated with sleep-disordered breathing [10]. Of increasing importance is the relationship between short sleep, obesity, and cardiometabolic outcomes [11,12]; nevertheless, little is known about these associations [13] or how public health interventions can be applied to prevent and treat adiposity and sleep-associated cardiometabolic disease risk factors [14].

Although progress has been made in modeling the relationship between sleep and adiposity, much work remains [15,16]. The data from the epidemiological and experimental studies of obesity and short sleep, although suggestive, need to be viewed within context. Epidemiological studies frequently have several methodological limitations, including being (1) retrospective, (2) cross-sectional, and (3) based on self-reported data [17]. Often, (4) nonvalidated measures [9] are used along with (5) inconsistent definitions of short sleep (eg, 6 [18] to 9 [19] hours). In addition, such studies have (6) limited control over potential confounding variables [20] and (7) report inconsistent modeling of sleep as a cause or consequence of obesity [21]. Taken together, these limitations often lead to mixed results. Moreover, they limit the ability to evaluate causal models linking adiposity and sleep duration, thereby precluding the capacity to posit sleep as a modifiable risk factor for the treatment of overweight and obesity.

Concurrently, experimental studies often rely on (1) small sample sizes [22] combined with (2) terse periods of observation [23] (3) in rigorously controlled settings (potentially eliciting Hawthorne effects [24]), and (4) the suppression of one or more free-living factors hypothesized to influence the relationship between adiposity and sleep (eg, prohibiting exercise [25]). Although experimental studies are methodologically rigorous and suggestive of a causal connection between adiposity and sleep, the design features often limit translation to free-living populations.

The SLUMBRx (Short Sleep Undermines Cardiometabolic Health) study will capitalize on the strengths of the literature and attempt to address salient limitations. The methods and findings from the epidemiological literature will be considered by using a standardized survey approach to provide a theoretical backdrop against which to contextualize results. The findings and methods from the experimental literature will be incorporated by collecting objective measures of adiposity, sleep, and cardiometabolic outcomes. Therefore, the proposed SLUMBRx research study can assist in filling important gaps in our understanding of the relationship between these health outcomes.

### Overarching Aim of the Proposed Research

The overarching aim of the SLUMBRx study is to acquire a more comprehensive understanding of the relationship among adiposity, short sleep, and cardiometabolic risk factors (Table 1). The relationship between adiposity and sleep duration will be investigated to facilitate the primary aim of the SLUMBRx study. First, although studies have identified associations between obesity and short sleep in adults, there are occasional inconsistencies in research findings, with some studies observing an inverse linear relationship, U-shaped association, or no significant relationship [15,26,27]. Second, sex-based differences in the relationship between adiposity and sleep will be evaluated. Studies within this domain have identified interesting, yet often, inconsistent findings [25,28-31]. However, few studies have examined sex-based variations in adiposity and sleep duration using rigorous measures of adiposity [25,28-31]. Third, the association between adiposity and sleep on cardiometabolic outcomes requires further investigation, particularly considering updated blood pressure guidelines [32]. Fourth, adiposity, sleep, and cardiometabolic outcomes are influenced by a myriad of upstream and downstream factors [33]. Contextualizing the adiposity-sleep connection from a socioecological perspective [34] is crucial to understanding the full scope of their relationship and impact on cardiometabolic disease.

**Table 1:** Hypotheses and methods of analysis.

Hypothesis	Method of analysis
Hypothesis 1: participants who are overweight and obese will demonstrate significantly shorter sleep relative to participants classified as normal weight	Analysis 1: continuous outcome sleep hours will be evaluated using a one-way ANOVA^a^ for BMI (3 levels: normal, overweight, and obese)
Hypothesis 2: male and female participants will demonstrate significantly different adiposity effects on sleep duration outcomes	Analysis 2: continuous outcome sleep hours will be evaluated using factorial ANOVA. The interaction effects between sex (2 levels: male and female) and BMI (3 levels: normal, overweight, and obese) will be examined
Hypothesis 3: participants who are overweight and obese will exhibit greater cardiometabolic risk outcomes compared with those who are normal weight; this relationship will be stronger in participants who are overweight and obese with short sleep duration	Analysis 3: continuous cardiometabolic risk factors are dependent variables. Sleep (2 levels: short and normal) and BMI (3 levels: normal, overweight, and obese) are independent variables. Factorial ANOVA will be used to evaluate the interaction effects as well as simple effects of sleep and obesity on cardiometabolic risk factors
Exploratory specific aim 4: to explore the role of socioecological factors (societal, social, and individual levels) as effect modifiers in the relationship between adiposity indices, sleep, and cardiometabolic outcomes. No formal hypothesis testing will occur for specific aim 4, because of sample size limitations	Analysis 4: descriptive statistics will provide important information regarding the impact of socioecological variables on sleep. Linear mixed models will be used to examine multilevel effects. Sleep hours is the dependent variable. Cardiometabolic (cardio), obesity, social, and societal factors are the independent variables

^a^ANOVA: analysis of variance.

### Specific Aims and Hypotheses

A community sample (N=160) of participants who are normal weight, overweight, and obese will be invited to participate in the proposed SLUMBRx study to accomplish these goals. Phase 1 of SLUMBRx will include measurement of adiposity indices and cardiometabolic outcomes in a clinical setting. Phase 2 of SLUMBRx will include sleep apnea screening and 1 week of daily sleep diaries and actigraphy using epidemiological data collection techniques. During phase 2, participants completed a set of validated questionnaires examining sociodemographic factors associated with adiposity indices, sleep disorders, and cardiometabolic outcomes.

Objective measures of adiposity indices, sleep outcomes, and cardiometabolic predictors will be collected from participants over the course of the SLUMBRx study. Specific measures will include the following:

- Adiposity indices: anthropometrics (BMI, skinfold thicknesses, and circumferences) [35,36] and air displacement plethysmography [37].
- Cardiometabolic predictors: blood pressure [38], pulse wave velocity and pulse wave analysis [39,40], and blood-based biomarkers [41].
- Sleep outcomes: home sleep testing or sleep apnea [42,43], sleep diaries [44], and actigraphy [45].

In addition to clinical outcomes, sex-based differences in the relationship between adiposity and sleep duration will be investigated [25,28-31]. This study will also assess socioecological factors [33] that influence adiposity indices, sleep, and cardiometabolic outcomes as an exploratory aim.

- Specific aim 1: to determine the relationship between adiposity and sleep duration.
  Hypothesis 1: participants who are overweight and obese will demonstrate significantly shorter sleep than participants classified as normal weight.
- Specific aim 2: to evaluate sex-based differences in the relationship between adiposity and sleep duration.
  Hypothesis 2: male and female participants will demonstrate significantly different adiposity effects on sleep duration outcomes.
- Specific aim 3: to assess whether adiposity and sleep duration influence cardiometabolic outcomes.
  Hypothesis 3: participants who are overweight and obese will exhibit greater cardiometabolic risk outcomes compared with participants who are of normal weight; this relationship will be stronger in participants who are overweight and obese with short sleep duration.
- Specific aim 4 (exploratory): to explore the role of socioecological factors as effect modifiers in the relationship between adiposity, sleep, and cardiometabolic outcomes.
  Owing to sample size limitations, formal hypothesis testing will not occur for aim 4.

## Methods

### Sample Size Considerations

Power analyses for testing the SLUMBRx hypotheses were calculated [46] using an αof .05 and effect sizes that would yield a power of 0.80. Sample size estimates were primarily based on detecting significance for *specific aim 1*. Data from the 2007 to 2008 National Health and Nutrition Examination Survey (NHANES) [47] were used to estimate the power analysis parameters. Analysis of NHANES data revealed that mean sleep hours were 7.08 (SD 1.3) hours for individuals who are normal weight, 6.84 (SD 1.3) hours for individuals who are overweight, and 6.72 (SD 1.3) hours for individuals who are obese. Although NHANES data were based on self-reports, the SLUMBRx study will apply validated sleep diaries and objective sleep measures (accelerometers). Therefore, the means and SDs for the proposed SLUMBRx study would likely be smaller than those of NHANES. Applying an SD of 0.60 with the same mean values of sleep duration as NHANES for the 3 groups, 53 participants per group are required to achieve a power of 0.80, with an effect size of 0.25 for a total sample size of 159 participants for *specific aim 1*. Similar sample sizes are required for the remaining specific aims. For example, using the same parameters, *specific aim 2* requires a sample size of 52 participants per group (156 total) to detect significant adiposity by sex interaction (0.26 effect size). A total of 160 participants will be recruited to accommodate specific aims 1 to 3.

A power analysis was not conducted for *exploratory specific aim 4*. Given that this is the first attempt at building a socioecological model of sleep, limited information is available to specify sample size parameters. Although formal hypothesis testing will not be conducted for *specific aim 4*, it is expected that the descriptive statistics and multilevel model will provide important information regarding the impact of socioecological variables on sleep. There has been a continuous call for the inclusion of socioecological models in public health, as these models can provide a more comprehensive context to understand sleep [48].

### Inclusion and Exclusion Criteria

Inclusion criteria will limit SLUMBRx study eligibility to (1) adults, 18 years of age or older, (2) with reliable internet access, email, and telephone, and (3) a commitment to completing all study activities. Exclusion criteria for the SLUMBRx study are described in Textbox 1.

**Exclusion criteria for the SLUMBRx (Short Sleep Undermines Cardiometabolic Health) study.**

- Participants must be willing to commit to the SLUMBRx protocol, which includes air displacement plethysmography, pulse wave velocity and analysis, collection of blood-based biomarkers, home sleep testing, and completion of questionnaires. Commitment will be assessed during the informed consent process to help ensure compliance and reduce study dropout.
- Unstable medical or psychological distress will be assessed using the Systematic Assessment for Treatment Emergent Events [49,50] questionnaire and the Kessler-6 Psychological Distress Scale [51] to help deter confoundment of lab-based assessments.
- Current medical interventions and medications will be assessed through self-report. Medical interventions via prescription medications (eg, statins) and treatment devices (eg, continuous positive airway pressure therapy) may confound the relationship between adiposity, sleep, and cardiometabolic disease. Similar approaches have been used in other research protocols [52].
- Evidence of substance abuse will be assessed by the CAGE (cut-annoyed-guilty-eye)-Adapted to Include Drugs [53] to help deter confoundment of lab-based assessments
- Pregnancy will be assessed by self-report to help ensure the fetus is not exposed to the procedures involved with the study and ensure the biopsychosocial changes that occur with pregnancy do not confound the lab-based assessments.
- Inadequate language comprehension will be assessed during the intake portion of the lab-based assessment to assure the quality of self-report data, as all the measures are in English
- Lack of return addresses will be assessed through self-report by asking potential participants to provide their mailing address. Data collection tools, such as sleep apnea home-testing kits, must be returned upon use. As such, a return mailing address is required.
- Self-report data will primarily be collected through the web-based data portal; therefore, reliable internet access is required. An email address and phone number are required to communicate with patients if they have any questions about the study and schedule the study's lab-based component.
- Underweight BMI will be assessed by calculating self-reported height and weight. Although underweight BMI is an important variable [54], budgetary constraints will prevent studying this issue for this proposal.
- Long sleep, defined as habitual sleep greater than 9 out of 24 hours [55], will be assessed using item 4 from the Pittsburgh Sleep Quality Index [56]. Long sleep is a proposed risk factor for obesity and cardiometabolic disease. However, long sleep likely interacts with obesity and cardiometabolic disease through unique mechanisms [57]. These issues, combined with budgetary constraints, informed the decision to exclude participants who are underweight from this study.

For screening purposes, BMI [35] will be calculated from self-reported height or weight. Although BMI will be recalculated in a clinical setting during phase 1 of this study, it is anticipated that measurement error associated with self-reported height and weight for BMI calculations will be acceptable for screening and initial group allocation purposes (normal weight, overweight, and obese). Demographic data will also be collected (eg, sex, age, race, or ethnicity) along with a request for a commitment to study completion. Filler items will be included in the screening survey to rule out professional participants. Consent will be obtained for the screening data.

### Inclusion of Children, Minorities, and Women

Children will not be included in the SLUMBRx study. Although childhood obesity and poor sleep in children are critical health concerns [58], the aims of SLUMBRx pertain specifically to adults. Risk factors for short sleep and obesity in children appear to operate differently than those underlying adult obesity and poor sleep [59]. Regarding minority enrollment, there will be an equal opportunity for members of all ethnic and racial backgrounds to participate in the SLUMBRx study. Study design and recruitment are expected to ensure that minorities are well represented. On the basis of the United States Census Bureau’s 2016 population estimates for Tuscaloosa [60], we expect our sample to reflect the following racial and minority representation: 65.1% White, 31.8% African American or Black, 1.5% Asian, 0.3% American Indian or Alaskan Native, and 0.1% Native Hawaiian or Pacific Islander. This area’s ethnic distribution is 3.5% Hispanic or Latino and 62.1% White alone, not Hispanic or Latino. Recruitment prioritizes a representative minority sample for this metropolitan area. Minority inclusion will be closely monitored during recruitment to ensure adequate representation. Prioritized recruitment of minority groups will be utilized should all groups be underrepresented.

Specific aim 2 of the SLUMBRx study is particularly focused on investigating sex-based differences in the relationship between adiposity and sleep duration. Therefore, the inclusion of women is paramount to the success of this proposal. Multiple studies suggest that women are at higher risk for obesity from short sleep than men [28,61,62]. Other studies have found that men with short sleep are at greater risk for obesity than women [25,29,30]. Ford et al [31] found that sleep duration was associated with BMI; however, there was no evidence of a statistical interaction by gender, race, or ethnicity.

### Possible Risks and Benefits to Participants

#### Risks

The risk to participants is expected to be minimal, as there are minimal risks associated with the completion of the web-based screening survey. Participants will be informed that they may withdraw from the SLUMBRx study at any time if they experience discomfort. Regarding air displacement plethysmography, mild discomfort because of the small dimensions of the chamber is possible. Participants who experience distress will be referred for appropriate treatment. There is minimal risk associated with the pulse wave velocity device as it is applied externally and is noninvasive. In terms of the blood draw, infection, bruising, fainting, and a small amount of bleeding are possible. As participants will be in a fasted state, there are some associated health risks, including dizziness, decreased alertness, and symptoms associated with low blood sugar levels. A qualified phlebotomist will draw blood following standard procedures. In the event that the participants have an adverse reaction to any of the medical procedures, the proximity of the clinical setting where SLUMBRx will transpire to proximity to the local hospital will permit rapid access to emergency response teams. Regarding the home sleep testing kit, mild discomfort from wearing the equipment is possible. To minimize risk, participants will be given a demonstration regarding the setup of the device and oral and illustrated instructions. In addition, a 24-hour contact number to call for any problems will be provided. Similarly, mild discomfort from wearing the blood pressure cuff is possible. To help offset risk, arm circumference will be measured to ensure an appropriate cuff size. There are minimal risks associated with the completion of the web-based screening survey. The primary risk pertains to confidentiality rather than safety risks. All participants will be assigned a study ID. Study ID numbers will be used in all documents for review, evaluation, and analysis. No verbal or written information concerning participants will be released without written consent from the subject. Publication of all results will maintain participant confidentiality. Only group data will be reported. Data will be stored under double-locked conditions and made available only to qualified staff working directly on the project.

#### Benefits

Participants may gain satisfaction knowing they contributed to a scientific study that may someday result in better diagnostic and treatment options for adiposity, sleep, and cardiometabolic risk factors. Participants will undergo health screening related to their body composition and sleep and be referred to a local sleep clinic if they have any medical questions or concerns. They will also be provided with a financial incentive and receive free food and drink during the conclusion of the lab-based portion of the study. All participants who complete the full study will be paid US $100.00; payment will be prorated as follows: US $40.00 for the lab-based component of the study (phase 1) and US $40.00 for the home-based component of the study (phase 2). A US $20.00 bonus will supplement the compensation for participants that provide complete data. However, the primary benefit for eligible participants will be access to health screening services.

## Results

### Recruitment

A community sample (N=160) of participants who are normal weight, overweight, and obese living in the city of Tuscaloosa, Alabama, will be invited to participate in the SLUMBRx study. Strategies for directing potential participants to the study include posting announcements to web-based classifiers, distribution of informational flyers or brochures throughout the community, including community centers (Tuscaloosa County Park and Recreation Authority), and churches, ads in minority papers (eg, Mobile Beacon and Alabama Citizen), and poster advertisements on the city commuter system (Tuscaloosa Transit Authority). Research study advertising networks established by the University of Alabama Strategic Communications department will also be used. Specific procedures will be employed to maximize our outreach to minority communities and aggregate an ethnically diverse and representative sample, such as targeted advertising, posting in places that serve minority populations, and working with local clergy. In addition to standard advertisement strategies, the study will also be advertised through the University of Alabama media and the established research networking channels developed and fostered by the University of Alabama Institute for Rural Health Research. Each advertisement medium will direct respondents to the main study page.

With these plans, modest recruitment targets, and the Tuscaloosa community’s size (population 99,543), it is anticipated that recruitment will be accomplished in a timely manner.

Census data [63] indicate that the crude prevalence of obesity for Tuscaloosa is 41.4%. Panel 3 provides model-based estimates of sleeping less than 7 hours among adults aged 18 years and older living in Tuscaloosa. Census data also indicate that the crude prevalence of short sleep for Tuscaloosa is 46.1%.

Respondents will be able to access the main study page and screening survey through a variety of entry points. For example, flyers will have removable tabs that provide the study page URL, which respondents may tear off and store on their personal computer. In addition to listing the website URL, paper mediums (eg, flyers) will include a quick response code, allowing respondents to access the study’s welcome page using their phone camera. The welcome page will provide a basic orientation for the study. If the subject is interested in participating in the study, they will be directed to click on the *I agree* button located at the bottom of the welcome screen. By clicking *I agree*, respondents will be directed to read and complete 2 informed consent documents, one related to the study and the other related to the Health Insurance Portability and Accountability Act (HIPAA). Once these are read, if the respondent wishes to continue in the study, they must agree to the stipulations of the study consent form and HIPAA documents by clicking on *I agree* button located at the bottom of the page. The informed consent documents will be protected by a content validation algorithm, ensuring that participants cannot proceed to the screening survey without first providing consent. Completing the consent documents initiates the assignment of a study ID, recording of the date and time, and administration of the screening survey. The screening survey will also capture general information such as the respondents’ names, mailing addresses, phone numbers, email, and demographic information.

### Screening

Eligibility for the SLUMBRx study will be determined by screening with standardized scales and questionnaires. This component of the study will allow for the profiling of participants in a manner that is consistent with large-scale surveys undertaken with epidemiological research. All potential participants may respond to the screening survey. BMI will be calculated using respondents’ self-reported height and weight, calculated as weight/height^2^ (expressed as kg/m^2^) [8]. Although BMI will be recalculated under controlled conditions during phase 1 of the study, we anticipate measurement error associated with self-reported height and weight for BMI calculations acceptable for screening purposes. On the basis of BMI, participants invited into the study will be (by self-report) of normal weight (BMI 18.5 to <25.0), overweight (BMI 25.0 to <30.0), or obese (BMI ≥30.0). For the purposes of this study, participants categorized as underweight (BMI <18.5) will be excluded from participating. Researchers have proposed different mechanisms (eg, eating disorders [54]) may be responsible for the sleep outcomes observed in this group. These issues, combined with budgetary constraints, informed the decision to exclude participants who are underweight from this study.

Following the completion of the screening survey, responses will be reviewed to determine eligibility for the SLUMBRx study. Eligibility was determined using the inclusion or exclusion criteria. As this study sought equivalent sample sizes for the 3 groups being investigated (normal weight, overweight, and obese), once a particular group has reached saturation (determined by calculating BMI from self-reported height and weight), respondents falling into a saturated group will be ineligible to participate in the study. Respondents meeting the study criteria will be contacted to establish a convenient date or time for initiation of the study’s laboratory-based portion. Participants will also be made a temporary parking pass to grant them convenient access to the Nutrition and Metabolism Research Lab.

### Consent Process

Potential participants will be required to provide consent before being given access to the screening survey (for survey data only). The SLUMBRx study will also require a consenting process. In this instance, informed consent will be obtained before initiating the laboratory-based study. In phase 1, participants will be given a copy of the consent form to read in private and the opportunity to ask questions. They will be informed that declining to participate will in no way interfere with their relationship with the University of Alabama and that participation is voluntary, and they may withdraw at any point. The principal investigator (PI) will take every precaution to ensure that prospective participants understand what is being asked of them before signing the consent form. In addition to the screening survey, no data will be collected without obtaining informed, signed, written consent.

### Data Storage, Confidentiality, and Privacy

Every effort will be made to ensure participant privacy. Data safety and monitoring will be evaluated by a data and safety monitoring board. Whenever feasible, identifiers are removed from the study-related information. All blood samples will be identified by a barcode generated by a computer in place of identifiable information. Data obtained for the proposed study will be used only for this research project. A review of informed consent documents for phases 1 and 2 of the SLUMBRx study will occur face-to-face with participants behind closed doors in a private interview room located within the Nutrition and Metabolism Research Lab. Lab-based data will be collected in patient rooms (behind closed doors with no windows in the room) located within the Nutrition and Metabolism Research Lab. All paper records will be kept in locked filing cabinets located within the PI’s locked office (doubly locked) and will only be accessible to personnel involved in the study. All electronic records will be stored at the University of Alabama’s password-protected HIPAA-compliant cloud service. Access to the cloud requires a password and confirmation from a second device using the Duo security platform. For example, when attempting to log on to the cloud, not only must the PI enter the correct password, but a confirmation of entry is sent to the PI’s mobile device for confirmation of access. The password to the cloud will frequently change as an additional form of data protection. All interactions, including phone calls and emails, to and from participants will comply with HIPAA regulations.

### Data Collection

Following a web-based screening survey of participants, qualified, consenting respondents will participate in a 2-phase SLUMBRx study. Phase 1 entails the collection of objective measures of adiposity and cardiometabolic outcomes. Phase 2 involves collecting subjective and objective sleep data using sleep diaries, actigraphy, and home sleep testing for sleep apnea. During phase 2, participants will also complete a battery of validated questionnaires and scales assessing socioecological factors associated with sleep, adiposity, and cardiometabolic outcomes.

#### Phase 1: Laboratory-Based Study

This component of the SLUMBRx study will allow for objective measures under controlled conditions typical of lab-based studies. Participants will be escorted to the Nutrition and Metabolism Research Lab, where they will review the study protocol and sign an informed consent form. Next, participants will undergo a battery of tests related to the measurement of adiposity and cardiometabolic indices outlined below.

##### Air-Displacement Plethysmography

The adult air-displacement plethysmography system, commercially produced under the trade name BOD POD (COSMED), has been validated [64,65] in adult populations and is considered robust for the measurement of human body composition [66,67]. Air-displacement plethysmography is a whole-body densitometric technique based on the displacement of air rather than water; it evaluates human body volume, body density, and body composition by measuring air displacement and the subsequent determination of body volume through the application of Boyle law. The air-displacement plethysmography unit consists of a dual-chamber plethysmograph, an electronic scale, and a computer. This equipment has a single structure containing 2 chambers separated by a device that produces pressure fluctuations and volume changes, permitting body volume assessment; this is followed by two to three 50-second measurements with the subject in the BOD POD using estimated thoracic lung volume to determine the fat mass, fat-free mass, and percentage body fat (%BF).

##### Anthropometrics

All measurements will adhere to standardized protocols, and anthropometric measures will all be performed by the same trained clinician. BMI will be calculated as body weight (in kilograms) without shoes and with light clothing, divided by height (in meters) squared—expressed as kg/m^2^). Body weight will be measured to 0.05 kg using the BOD POD electronic scale. Height will be measured to the nearest 1 mm using a stadiometer (Seca). For both height and weight, 2 measurements will be obtained and averaged; a third measurement will be obtained if the first two are more than 0.5 cm/0.1 kg apart and then averaged. Skinfold thicknesses will be measured to the nearest 0.1 mm with a skinfold caliper at the following sites using the same averaging protocol: (1) triceps, halfway between the acromion process and the olecranon process; (2) biceps, at the same level as the triceps skinfold, directly above the center of the cubital fossa; (3) subscapular, 20 mm below the tip of the scapula, at an angle of 45° to the lateral side of the body; and (4) suprailiac, 20 mm above the iliac crest and 20 mm toward the medial line. Circumferences will be measured with a nonelastic tape within 1 mm as follows: (1) waist circumference will be measured at the end of a gentle expiration midway between the lowest rib and iliac crest and (2) hips will be measured at the greater trochanter. In addition to standard circumference data, neck circumference will be measured because of its correlation with cardiometabolic risk [68] and sleep apnea in short sleepers [69]. Therefore, (3) neck circumference will be measured in the midway of the neck between the midcervical spine and midanterior neck. All skinfold and circumference measures will be taken with the participants standing upright, with the face directed toward the clinician, and shoulders relaxed.

##### Pulse Wave Velocity and Pulse Wave Analysis

Arterial stiffness will be assessed by radial artery applanation tonometry recording (Mobil-O-Graph [70]). Pulse wave analysis is a noninvasive, valid, reliable, and inexpensive technique that offers significant clinical and epidemiological potential. Briefly, pressure is applied to the radial artery by a probe placed on the wrist. The radial wave created by the signal is used to generate an aortic pulse pressure waveform, which is used to derive an augmentation index (AI_x_). AI_x_ is an indication of arterial stiffness, with a higher AI_x_ signifying greater arterial stiffness [39,40].

##### Blood Pressure

Hypertension has been proposed as an intermediate step in the causal pathway between decreased sleep duration and cardiovascular disease [57]. During the same visit, 2 systolic and diastolic blood pressure readings will be recorded (Mobil-O-Graph [71]) with a 5-minute interval and averaged for analysis. A third measurement will be obtained if the difference between the first two is >5 mm Hg [72]. Both blood pressure and pulse wave velocity or pulse wave analysis will be measured in a seated position after a 10-minute rest period in a quiet room.

##### Cardiometabolic Biomarkers

Sleep duration is hypothesized to influence cardiometabolic biomarkers [73]. Hyperlipidemia is an established risk factor for cardiovascular disease [74] and may be linked to short sleep [41]. Blood samples will be collected by a trained phlebotomist in the Nutrition and Metabolism Research Lab for measures of cholesterol (lipid panel with low-density lipoprotein:high-density lipoprotein ratio) and glucose [52]. Samples will be taken in a sitting position, centrifuged immediately, and transferred under cold chain conditions to a central laboratory for analysis.

#### Phase 2: Prospective Assessment

This component of the SLUMBRx study will allow for the sampling of adiposity, sleep, and cardiometabolic outcomes in a manner consistent with naturalistic or ambulatory studies.

##### Home Sleep Testing

During their visit to the Nutrition and Metabolism Research Lab, participants will be provided with a home sleep recording device that will be used to screen for sleep apnea by recording airflow, respiratory effort, and oxygen saturation. Participants will be instructed on the device’s use and will be given a prepaid envelope to return the device. All recordings will be scored by trained technicians at the Alabama Neurology and Sleep Medicine. Participants with an apnea-hypopnea index of ≥5 will be referred for treatment [75].

##### Actigraphy and Questionnaires

Participants will complete a battery of questionnaires through a web-based data portal. Questionnaires will include the Sleep Timing Questionnaire (STQ) for habitual sleep schedule [76], the Epworth Sleepiness Scale (ESS) for subjective sleepiness [77], and the Pittsburgh Sleep Quality Index (PSQI) [56] for sleep quality. Participants will also complete sleep diaries [44] and wear an accelerometer for 1 week to provide an objective assessment of sleep, following the guidelines by Ancoli-Israel et al [78,79]. These assessments will be used to corroborate the retrospective measures of sleep duration. In addition, previously validated questionnaires and scales representing salient levels of the **socioecological** model of sleep (societal, social, and individual) developed by Grandner et al [12,33] will be completed by participants. Examples of questionnaires include societal level [80,81] (Barriers to Care Questionnaire [82]), social level [83,84] (Job Satisfaction Survey [84]), and individual level [85,86] (Healthy Literacy Questionnaire [87], Self-Efficacy for Sleep Scale [88], and demographics such as education, marital status, annual household income, etc).

##### Potential Problems and Alternatives

If web-based data collection presents a significant barrier, especially as it pertains to the recruitment of women and/or minorities, paper-and-pencil versions of the data collection materials will be provided to respondents. An alert system will be incorporated into the web-based data portal to assist with compliance, alerting study staff if a questionnaire or diary entry has been missed. In addition, participants will be provided a 24/7 contact number and directions that will be tested for readability. Budgetary allowances for 10 additional participants (6.25%) were allocated as safeguards against attrition. Records of respondents’ baseline characteristics will determine whether differences between those that participate and those that do not participate will be retained. If differences are identified, statistical adjustments will be made to account for possible selection bias. If violations to model assumptions are detected, problematic data will be inspected. If possible, model modifications (eg, transformation) will be made, rather than dropping cases. The risks involved in this study are minimal. Protocols will be enacted to manage potential risks.

### Data Analytic Strategy and Analysis

#### Data Collection and Management

Most of the self-report data will be collected via a web-based portal (Qualtrics LLC) dedicated to the proposed study. Data from other sources will be entered into the same system by trained research staff. When the data are fully aggregated, they will be downloaded, screened, and summarized. A final analysis database will be constructed with all summary variables deidentified according to standardized confidentiality procedures.

#### Data Analysis Plan

The primary study variables for the SLUMBRx study include (1) sleep hours, (2) systolic blood pressure, (3) diastolic blood pressure, (4) apnea-hypopnea index, (5) augmentation index, (6) total lipid levels, and (7) glucose. It is hypothesized that, relative to participants who are normal weight, those who are overweight and obese will have differences in the distribution of the primary study variables reflected either in mean changes and/or increased variance. The primary study hypotheses will be tested using analysis of variance (ANOVA). ANOVA models utilize all available outcome data in the estimation of group differences for individual outcomes. Due to the potential correlation among cardiometabolic variables, a multivariate ANOVA omnibus test will be calculated to control for familywise type I error rate and assess patterns between multiple dependent variables. Then, univariate ANOVA for each dependent variable will be conducted as follow-up tests. A series of secondary outcome variables will also be analyzed using ANOVA models to examine group differences for a constellation of variables (eg, sleep quality, %BF, stroke volume, neck circumference, mean arterial pressure, etc). Specific aim 4 is exploratory in nature. Due to the sample sizes required to build robust multilevel models, formal hypothesis testing will not be conducted.

#### Treatment of Missing Data

A multiple imputation approach will be used in the event of missing data [89]. Under this procedure, we will use SAS PROC.MI to complete data sets and combine these using SAS PROC.MIANALYZE to produce final inferential results. Sensitivity analysis will be conducted by comparing results from only complete cases and data imputed [90]. Table 1 summarizes the hypotheses, methods of analysis, and statistical models used for the SLUMBRx study. If the multiple imputation computation system fails to work correctly, then nonparametric analysis methods will be applied.

## Discussion

### Principal Findings

Obesity and short sleep duration are highly prevalent, interconnected risk factors for cardiometabolic disease in adults; however, a comprehensive adiposity-sleep model has remained elusive because of the complexity of the relationship between these 2 health-related states. The SLUMBRx study can help to drive preventative public health interventions by (1) probing the validity of the adiposity-sleep hypothesis, (2) modeling upstream and downstream demographic and ecological factors of adiposity and sleep, and (3) determining whether adiposity and sleep influence cardiometabolic disease risk factors. The proposed research will form the background of new lines of epidemiological and experimental inquiry seeking to unpack the relationship between adiposity, sleep, and cardiometabolic disease. Furthermore, this study will provide data necessary to propose and design public health interventions for obesity and short sleep duration by identifying appropriate measures to be used as intervention endpoints.

### Conceptual Innovations

SLUMBRx is conceptually innovative in at least five ways. First, this proposal explores the potential of adiposity indices as modifiable risk factors for short sleep. If participants who are overweight and obese demonstrate shorter sleep than those who are of normal weight, it will lend credence to the hypothesis proposed in specific aim 1 that adiposity independently and/or interactively contributes to short sleep. Second, few studies have used rigorous adiposity measurement when investigating sex-based differences between sleep and adiposity as delineated in specific aim 2. Studies that have been conducted frequently produce conflicting or null results, suggesting that additional research is needed to understand this phenomenon. Third, sleep, adiposity, and cardiometabolic data will be collected predominantly from participants in underserved, rural settings (Tuscaloosa county in Alabama), with an anticipated high prevalence of short sleep (<7 hours, estimated at 46.1%) and obesity (estimated at 41.4%) [91]. Data collection in rural settings will inform recruitment strategies for future studies. Fourth, sleep assessment will be conducted both retrospectively (with the STQ [76], ESS [77], and PSQI [56]) and prospectively (home sleep testing, sleep diaries, and actigraphy). Retrospective assessment using the STQ represents one of the first uses of a validated instrument to assess sleep schedules for identifying short sleepers. Prospective assessment, which is rarely done, is also important because it allows for determining whether self-reports of short sleep are reliable over time. Fifth, the data collected as part of specific aim 4 is innovative as research investigating upstream and downstream **socioecological** drivers of sleep is limited [20,78].

### Methodological Innovations

The SLUMBRx investigation is also methodologically innovative in several ways. First, this proposal has the potential to drive public health intervention research by probing the validity of the obesity-sleep hypothesis using objective sleep and adiposity metrics. Second, the influence of sleep duration and adiposity indices on cardiometabolic outcomes, as detailed in specific aim 3, is a novel extension of the epidemiological and experimental body of research. Third, this project will assess the feasibility of public health sleep-based research undertaken in underserved, rural communities. It is also expected that the implementation of the protocol will inform recruitment strategies for future grants built off this proposal. Fourth, the data collected from this study will assist in proposing and designing future public health interventions by identifying clinical endpoints for sleep, adiposity, and cardiometabolic outcomes. Fifth, this study will use a web-based data portal to provide convenient access to study materials for participants. In addition, the data portal will employ time stamping (assuring prospective data are indeed prospective) and content validation of response options (eg, restricting out-of-range responses). The system also eliminates data entry and transcription errors that can occur when transcribing paper-and-pencil data into electronic spreadsheets.

### Conclusions

Obesity and short sleep duration in adults comprise prominent public health concerns because of their high prevalence and association with a wide range of harmful sequelae, including premature mortality [92], cardiovascular disease [3], and diabetes [93]. In recent years, there has been increased interest in the potential link between obesity and short sleep [11,13,26,94-96]. The proposed SLUMBRx investigation will contribute to developing a comprehensive adiposity-sleep model while also laying the groundwork for a future research program that aspires to prevent and treat adiposity and sleep-associated cardiometabolic disease risk factors.

## Appendix

Multimedia Appendix 1 Funded grant peer-review documentation.

## Abbreviations

%BF: and percentage body fat
AIx: augmentation index
ANOVA: analysis of variance
ESS: Epworth Sleepiness Scale
HIPAA: Health Insurance Portability and Accountability Act
NHANES: National Health and Nutrition Examination Survey
PI: principal investigator
PSQI: Pittsburgh Sleep Quality Index
SLUMBRx: Short Sleep Undermines Cardiometabolic Health
STQ: Sleep Timing Questionnaire
