# Supplementary material for: Modeling Risk Factors for Sleep- and Adiposity-Related Cardiometabolic Disease: Protocol for the Short Sleep Undermines Cardiometabolic Health (SLUMBRx) Observational Study
Source: JMIR Res Protoc. 2021 Mar 9;10(3):e27139. doi: 10.2196/27139 (PMC7988396; doi:10.2196/27139)
Supplement: Multimedia Appendix 1 [file resprot_v10i3e27139_app1.pdf]

SUMMARY STATEMENT
(Privileged Communication)

PROGRAM CONTACT:
Jared Reis

Release Date: **04/26/2019**
Revised Date:

Application Number: **1 K01 HL145128-01A1**

Principal Investigator

KNOWLDEN, ADAM

Applicant Organization: UNIVERSITY OF ALABAMA IN TUSCALOOSA

Review Group: **MCBS (MA)**
NHLBI Mentored Clinical and Basic Science Review Committee
NHLBI Mentored Clinical and Basic Science Review Committee

Meeting Date: **03/07/2019**
Council: **MAY 2019**
Requested Start: **12/01/2019**
RFA/PA: **PA18-369**
PCC: **SHEF N**

Project Title: **Adiposity and Sleep-Associated Cardiometabolic Disease Risk Factors**

SRG Action: **Impact Score:20**
Next Steps: **Visit https://grants.nih.gov/grants/next_steps.htm**
Human Subjects: 30-Human subjects involved - Certified, no SRG concerns
Animal Subjects: 10-No live vertebrate animals involved for competing appl.
Gender: 1A-Both genders, scientifically acceptable
Minority: 1A-Minorities and non-minorities, scientifically acceptable
Age: 3A-No children included, scientifically acceptable

Project Year	Direct Costs Requested	Estimated Total Cost
1	133,834	144,541
2	145,089	156,696
3	153,004	165,244
4	159,549	172,313
5	165,556	178,800
TOTAL	757,032	817,595

ADMINISTRATIVE BUDGET NOTE: The budget shown is the requested budget and has not been adjusted to reflect any recommendations made by reviewers. If an award is planned, the costs will be calculated by Institute grants management staff based on the recommendations outlined below in the COMMITTEE BUDGET RECOMMENDATIONS section.

1K01HL145128-01A1 Knowlden, Adam

NHLBI "K" series resubmission/amended application due date is Mar. 12, July 12, or Nov. 12. In addition, a Letter of Intent (LOI) is requested from the applicants who are planning to submit a resubmission application. The LOI should be submitted one month in advance. Submit LOI dates are February 12, June 12, or October 12. Submit LOI to nhlbichiefreviewbranch@nhlbi.nih.gov

Note: New NIH Policy on Resubmission (amended) Applications
Please see the following link for clarification: http://grants.nih.gov/grants/guide/notice-files/NOT-OD-09-003.html

Please be advised that NIH has adopted a **new policy** for accepting **Post-Submission Application Materials.** Please see the following link and related notices for a description of acceptable and unacceptable post-submission application materials: http://grants.nih.gov/grants/guide/notice-files/NOT-OD-10-091.html

CRITIQUES
The comments in the CRITIQUE section were prepared by the reviewers assigned to this application and are provided without significant modification or editing by staff. They are included to indicate the range of comments made during the discussion and may not reflect the outcome. The RESUME AND SUMMARY OF DISCUSSION section summarizes the final opinion of the committee after discussion and is the basis for the assigned priority score.

RESUME AND SUMMARY OF DISCUSSION: This outstanding, amended application was submitted in response to the Mentored research scientist career development award announcement. The application is well organized and the applicants provide a good and thoughtful response to the prior critiques.

The candidate and the institutional commitment are both outstanding to exceptional. The mentoring team is outstanding. The team is now more streamline and the team members grant portfolios are now clearly described. How the team will be managed is poorly addressed. The career development plan excellent to exceptional. The plan is solid and feasible.

The applicants "seek to contribute to the development of a comprehensive adiposity-sleep model, while laying the groundwork for a future program of research seeking to prevent and treat adiposity and sleep-related cardiometabolic disease risk factors." The research plan is good to excellent. Only some of the reviewers are clear on how the research plan will lead to the candidate's future success. The applicants did not convince some of the reviewers that they can move from studying an association to studying causality. It is unclear whether the applicants' finds will have a clinical impact.

DESCRIPTION (provided by applicant): Background. Obesity and short sleep duration are significant public health issues with evidence suggesting these conditions are associated with premature mortality, cardiovascular disease, metabolic syndrome, and inflammation. In the United States, 37.9% of adults are classified as obese, having a body mass index (BMI) of 30 kg/m2 or greater. Stratified by sex, 35.2% of men and 40.5% of women meet the BMI cut-point for obesity. Similarly, curtailed sleep duration is commonplace, with an estimated 35.3% of adults in the United States receiving less than the recommended 7 hours of sleep during a 24-hour period. In recent years, there has been an increased interest in the potential link between obesity and short sleep duration due to: (1) the apparent parallel increase in prevalence of both conditions over the past few decades; (2) their overlapping association with cardiometabolic outcomes; and (3) the potential causal connection between the two health issues. Research. The proposed investigation will seek to contribute to the development of a comprehensive adiposity-sleep model, while laying the groundwork for a future program of research

seeking to prevent and treat adiposity and sleep-related cardiometabolic disease risk factors. The study proposed within this K-award is intended to investigate four topics pertinent to the adiposity-sleep hypothesis: (1) the relationship between adiposity and sleep duration; (2) sex-based differences in the relationship between adiposity and sleep duration; (3) influence of adiposity indices and sleep duration on cardiometabolic outcomes; and (4) the role of socioecological factors as effect modifiers in the relationship between adiposity indices, sleep, and cardiometabolic outcomes. To address these aims, the proposed study will employ a large-scale survey (n=1,000) to recruit 159 subjects (53 normal weight, 53 overweight, and 53 obese) to be assessed in two phases. Phase 1, an in-lab study, will be used to gather objective adiposity indices (air displacement plethysmography and anthropometrics) and cardiometabolic data (blood pressure, pulse wave velocity and pulse wave analysis, and blood-based biomarker). Phase 2, a one-week, home-based study, will be used to gather sleep-related data (home sleep testing/sleep apnea, actigraphy, sleep diaries). During Phase 2, detailed demographic and socioecological data will be collected to contextualize hypothesized adiposity and sleep-associated cardiometabolic disease risk factors. Collection and analyses of these data will provide necessary information to customize future observational and intervention research. Training. Pedagogically, the training plan for the K01 is comprised of the didactic training (coursework, seminars, symposia), mentoring (directed research, readings, tutorials), and dissemination deliverables (publications, presentations, and proposals) required to complete the proposed project and to initiate a pathway towards research independence. This career development plan builds upon the applicant's previous obesity and sleep health research and couples these two independent health issues into an interconnected line of inquiry.

PUBLIC HEALTH RELEVANCE
Obesity and short sleep duration are highly prevalent, interconnected risk factors for cardiometabolic disease in adults; however, a comprehensive adiposity-sleep model has remained elusive due to the complexity of the relationship between these two health-related states. The training and research proposed in this career development award will form the background of new lines of epidemiological and experimental inquiry seeking to unpack the relationship between adiposity, sleep, and cardiometabolic disease. This stream of research holds substantial promise for public health; objective, empirical data regarding the interaction between adiposity and sleep, contextualized within a socioeconomic framework, are important for understanding the pathogenesis of cardiometabolic disease and for developing public health interventions to prevent its conception and treat its consequences.

CRITIQUE 1

Candidate: 2
Career Development Plan/Career Goals /Plan to Provide Mentoring: 3
Research Plan: 3
Mentor(s), Co-Mentor(s), Consultant(s), Collaborator(s): 2
Environment Commitment to the Candidate: 2

Overall Impact: The candidate's potential for a productive, independent scientific research career is excellent. The level of responsiveness to the comments from the prior review was high and responses were thoughtful. The candidate proposes to investigate four topics pertinent to the adiposity-sleep hypothesis: (1) the relationship between adiposity and sleep duration; (2) sex-based differences in the relationship between adiposity and sleep duration; (3) influence of adiposity indices and sleep duration on cardiometabolic outcomes; and (4) the role of socioecological factors as effect modifiers in the relationship between adiposity indices, sleep, and cardiometabolic outcomes. The study will use a large-scale survey (n=1,000) to recruit 159 subjects (53 normal weight, 53 overweight, and 53 obese) to be assessed in two phases. Phase 1, an in-lab study, will be used to gather objective adiposity indices (air displacement plethysmography and anthropometrics) and cardiometabolic data (blood

pressure, pulse wave velocity and pulse wave analysis, and blood-based biomarker). Phase 2, a one-week, home-based study, will be used to gather sleep-related data (home sleep testing/sleep apnea, actigraphy, sleep diaries). The training plan includes didactic training (coursework, seminars, symposia), mentoring (directed research, readings, tutorials), and dissemination (publications, presentations, and proposals). This career development plan builds upon the applicant's previous obesity and sleep health research and couples these two independent health issues into an interconnected line of inquiry.

1. Candidate:
Strengths
- Resubmission: candidate. There is a solid publication record although some manuscripts may not be indexed in PubMed.
- Candidate is a tenure-track Assistant professor, trained in health promotion and education (Masters and PhD).
- The candidate is well poised to develop as an independent and productive researcher; and the candidate's prior training and research experience are appropriate for a K-award.
- The candidate indicates robust commitment to meeting the training and research objectives that would support development into an independent investigator.

2. Career Development Plan/Career Goals & Objectives:
Strengths
- Better explanation of the nature of the courses planned improves my confidence in its feasibility.

Weaknesses
- None.

3. Research Plan:
Strengths
- In response to prior critique, has incorporated a plan to address the possibility of needing to re-assign people into a new weight category if laboratory results differ from self-report measures.
- In response to the prior critique, conducted power analyses to understand level of power available for covariates and interactions.

Weaknesses
- Minor: Study is small and unable to investigate important populations, but this is understandable given the resources associated with the K-award.

4. Mentor(s), Co-Mentor(s), Consultant(s), Collaborator(s):
Strengths
- Important adjustments to the mentoring team making it more streamlined with appropriate expertise – Drs. Higginbotham, Grandner, and Allegrante will be the primary mentoring team with others providing supplementary advising. Figure 2 is a very helpful depiction of these changes.
- Grants portfolio of mentoring team clearly described.

Weaknesses
- None.

5. Environment and Institutional Commitment to the Candidate:
Strengths
- Strong and clear commitment at University of Alabama.
- High quality environment with equipment and resources needed to complete the study.
- Institution is clearly committed to candidate.

Weaknesses
- None.

Study Timeline:
Strengths
- Appropriate.

Weaknesses
- None.

Biohazards:
Not Applicable (No Biohazards).

Resubmission:
- Comments/critiques addressed thoughtfully.

Select Agents:
Not Applicable (No Select Agents).

CRITIQUE 2

Candidate: 2
Career Development Plan/Career Goals /Plan to Provide Mentoring: 1
Research Plan: 3
Mentor(s), Co-Mentor(s), Consultant(s), Collaborator(s): 2
Environment Commitment to the Candidate: 1

Overall Impact: This is a revised application for a PhD investigator who wishes to become an independent investigator in the study of adiposity and sleep-associated cardiometabolic disease risk factors. The mentorship plan is outstanding, although attention to how to coordinate the number of mentors and advisors is lacking. Overall, the applicant has been very responsive to critiques. Some minor concerns remain.

1. Candidate:
Strengths
- PhD Trained researcher with expertise in the area.
- Strong publication record.

Weaknesses
- Given the publication record and the expertise, could wonder if K mechanism is beneficial.

2. Career Development Plan/Career Goals & Objectives:
Strengths
- The specific aims are matched to training goals.
- The career plan has clarified that coursework is all possible through summer in response to ambitious training plan.
- Overall, this is a very well-organized written plan for career training.

Weaknesses
- None.

3. Research Plan:
Strengths
- The aims seem feasible in scope for a K award and relevant for this training plan.
- Overall has been very responsive to critiques of research plan such as including NHANES as a comparator.

- A more clear understanding of innovation is also described to highlight why this study will have impact.

Weaknesses
- Hard to understand if causal relationship investigated.
- While transition to R01 is mentioned, hard to understand what the R01 from this work would be.

4. Mentor(s), Co-Mentor(s), Consultant(s), Collaborator(s):
Strengths
- The candidate has made adjustments to mentorship team based on revision.
- The candidate has assembled an outstanding mentorship team with complementary expertise and some evidence of having worked together before.

Weaknesses
- The candidate still has a lot of mentors and advisors without much attention to how to coordinate conflicting guidance from so many senior investigators.

5. Environment and Institutional Commitment to the Candidate:
Strengths
- 75% protected time dedicated to mentor.
- Also comments on other support (IT, space, etc.) and release from teaching.

Weaknesses
- None.

Biohazards:
Not Applicable (No Biohazards).

Resubmission:
- Has been responsive to comments.

CRITIQUE 3

Candidate: 1
Career Development Plan/Career Goals /Plan to Provide Mentoring: 2
Research Plan: 5
Mentor(s), Co-Mentor(s), Consultant(s), Collaborator(s): 2
Environment Commitment to the Candidate: 1

Overall Impact: This application is a resubmission from a candidate trained in Health Promotion & Education who is already a tenure-line Assistant Professor. His goals are to develop expertise in the study of adiposity and sleep-associated cardiometabolic disease risk factors to build on his recent experiences conducting research documentation relationship between sleep quantity and health outcomes including obesity. The candidate is very good to outstanding from the perspective of publication record, career development plan, mentors, and institutional commitment. He has been responsive to the initial critiques. The main weakness is the hypotheses being tested in the research plan where causality may still be very challenging to determine even if the study is well powered. Also, the clinical impact of the additional information gained is questionable in terms of general recommendations related to healthy living. Nevertheless, the exercise of conducting an observational study is a valuable one and the data collected may still serve as good preliminary data larger scale studies when the candidate transitions to independence.

1. Candidate:
Strengths

- Non-science background and exposure to public health policy prior to shifting towards academic career.
- Strong letters of support.
- Very strong publication record since 2012 with majority being first author.
- Excellent potential for independence. In fact, independence has already been demonstrated in many ways.
- Multiple internally funded grants to support his research agenda to date.
- Multiple awards for research with broad media coverage of his work.
- Extensive experience in dissemination/implementation of prior studies he has been involved.

Weaknesses

- (Minor) Is next best step a 5-year K vs. an R series award?

2. Career Development Plan/Career Goals & Objectives:

Strengths

- Well organized career development plan in the context of proposed research and training needed to carry out that research.
- Responsive to suggestions by reviewers with initial submission.

Weaknesses

- Consider accelerating time to submit initial R01.
- Would benefit from attending focused courses/seminars in using Mendelian Randomization for causal inference given the research topic proposed.

3. Research Plan:

Strengths

- Provides the candidate with hands on experience conducting clinical observational research with the plan to enroll a new cohort and collect objective “gold-standard” measures of adiposity, sleep duration, and cardiometabolic risk factor data seems feasible (n = 160).
- Integration of multidimensional data.
- Inclusion of subjects from predominantly underserved, rural settings.
- Very clearly outline analytic plan that is easily achievable.
- These data may serve as excellent preliminary data for funding of a larger scale study.

Weaknesses

- Unmeasured factors may confound relationship between adiposity and sleep duration even among individuals without sleep apnea.
- Relationship between obesity and CMD is well-established and study may be too small and confounded to identify sleep duration as a modifier of this relationship.
- Other resources like the UK Biobank may provide more reliable evidence for Aims 1 and 2 hypotheses with more power and with more certainty in terms of causality using bi-directional Mendelian Randomization (genetic instruments of sleep duration and adiposity).
- Potential Low impact: not sure whether general established recommendations to maintain a healthy weight (e.g. BMI 18 to 25) and to obtain a healthy amount of sleep (on average 8 hours) will be impacted by these relationships.
- Multiple sections of proposal have been transferred to section 12. Protection of human subjects including Sample size/ power, recruitment, exclusion/inclusion criteria.

4. Mentor(s), Co-Mentor(s), Consultant(s), Collaborator(s):

Strengths

- Impressive and committed group of mentors and advisors covering all disciplines related to candidate expertise and career development.
- Adjustments responsive to suggestions with initial submission of proposal.

Weaknesses

- Lack of expertise in genetic epidemiology which can potential be a very useful adjunct in examining the relationship between obesity, sleep duration, and cardiometabolic disease.

5. Environment and Institutional Commitment to the Candidate:
Strengths
- Already has a strong commitment to candidate as he is on the tenure track as Assistant Professor since 2013.
- Statement that he will be able to increase his commitment to research training from 40 to >75%
- Equipment to conduct study appears to be in place.

Biohazards:
Not Applicable (No Biohazards).

Resubmission:
- See review above.

Select Agents:
Not Applicable (No Select Agents).

(End of Reviewers' Comments)

THE FOLLOWING SECTIONS WERE PREPARED BY THE SCIENTIFIC REVIEW OFFICER TO SUMMARIZE THE OUTCOME OF DISCUSSIONS OF THE REVIEW COMMITTEE, OR REVIEWERS' WRITTEN CRITIQUES, ON THE FOLLOWING ISSUES:

PROTECTION OF HUMAN SUBJECTS: ACCEPTABLE

INCLUSION OF WOMEN PLAN: ACCEPTABLE

INCLUSION OF MINORITIES PLAN: ACCEPTABLE

INCLUSION OF CHILDREN PLAN: ACCEPTABLE

VERTEBRATE ANIMALS: ACCEPTABLE

COMMITTEE BUDGET RECOMMENDATIONS: The budget was recommended with the following request.
The applicants should be asked to reconcile the discrepancy between the domestic travel costs of $19,200 in the budget justification and the cumulative domestic travel costs of $47,860.

TRAINING IN THE RESPONSIBLE CONDUCT OF RESEARCH (Resume): ACCEPTABLE

RESOURCE SHARING PLAN (Resume): UNACCEPTABLE
A plan was not included in this application.

AUTHENTICATION OF KEY BIOLOGICAL AND/OR CHEMICAL RESOURCES: NOT APPLICABLE

Footnotes for 1 K01 HL145128-01A1; PI Name: Knowlden, Adam

NIH has modified its policy regarding the receipt of resubmissions (amended applications). See Guide Notice NOT-OD-14-074 at http://grants.nih.gov/grants/guide/notice-files/NOT-OD-14-074.html. The impact/priority score is calculated after discussion of an application by averaging the overall scores (1-9) given by all voting reviewers on the committee and multiplying by 10. The criterion scores are submitted prior to the meeting by the individual reviewers assigned to an application, and are not discussed specifically at the review meeting or calculated into the overall impact score. Some applications also receive a percentile ranking. For details on the review process, see http://grants.nih.gov/grants/peer_review_process.htm#scoring.

MEETING ROSTER

NHLBI Mentored Clinical and Basic Science Review Committee
Heart, Lung, and Blood Initial Review Group
NATIONAL HEART, LUNG, AND BLOOD INSTITUTE
NHLBI Mentored Clinical and Basic Science Review Committee

MCBS (MA)
03/07/2019 - 03/08/2019

Notice of NIH Policy to All Applicants: Meeting rosters are provided for information purposes only. Applicant investigators and institutional officials must not communicate directly with study section members about an application before or after the review. Failure to observe this policy will create a serious breach of integrity in the peer review process, and may lead to actions outlined in NOT-OD-14-073 at https://grants.nih.gov/grants/guide/notice-files/NOT-OD-14-073.html and NOT-OD-15-106 at https://grants.nih.gov/grants/guide/notice-files/NOT-OD-15-106.html, including removal of the application from immediate review.

CHAIRPERSON(S)

ORTIZ, LUIS ALBERTO, MD
PROFESSOR
DIVISION OF OCCUPATIONAL & ENVIRONMENTAL MEDICINE
DEPARTMENT OF ENVIRONMENTAL/OCCUPATIONAL HEALTH
GRADUATE SCHOOL OF PUBLIC HEALTH
UNIVERSITY OF PITTSBURGH
PITTSBURGH, PA 15261

MEMBERS

ANDERSON, CHERYL ANN MARIE, PHD
ASSOCIATE PROFESSOR
DIVISION OF PREVENTIVE MEDICINE
DEPARTMENT OF FAMILY AND PREVENTIVE MEDICINE
SCHOOL OF MEDICINE
UNIVERSITY OF CALIFORNIA SAN DIEGO
LA JOLLA, CA 92093

ARDEHALI, REZA, MD, PHD *
DIVISION OF CARDIOLOGY
RONALD REAGAN UCLA MEDICAL CENTER
LOS ANGELES, CA 90095

ARORA, VINEET, MD *
ASSOCIATE PROFESSOR OF MEDICINE
UNIVERSITY OF CHICAGO
CHICAGO, IL 60637

ASSIMES, THEMISTOCLES LEONARD, MD, PHD *
ASSOCIATE PROFESSOR OF MEDICINE
STANFORD UNIVERSITY SCHOOL OF MEDICINE
STANFORD, CA 94305

BARRETT, NORA AMANDA, MD *
ASSOCIATE PHYSICIAN
BRIGHAM AND WOMEN'S HOSPITAL
ASSISTANT PROFESSOR OF MEDICINE
HARVARD MEDICAL SCHOOL
CHESTNUT HILL, MA 02467

BASTARACHE, JULIE ANNE, MD *
ASSOCIATE PROFESSOR
DIVISION OF ALLERGY, PULMONARY,
AND CRITICAL CARE MEDICINE
DEPARTMENT OF MEDICINE
VANDERBILT UNIVERSITY MEDICAL CENTER
NASHVILLE, TN 37232

CAI, CHENLENG, PHD *
PROFESSOR OF PEDIATRICS
PED-CARDIAC DEV BIOLOGY WELLS
SCHOOL OF MEDICINE
INDIANA UNIVERSITY
INDIANAPOLIS, IN 46202

CONROY, MARGARET B, MD, MPH
PROFESSOR
DIVISION OF GENERAL INTERNAL MEDICINE
DEPARTMENT OF INTERNAL MEDICINE
UNIVERSITY OF UTAH SCHOOL OF MEDICINE
SALT LAKE CITY, UT 84112

CURTIS, J RANDALL, MD *
PROFESSOR
DIVISION OF PULMONARY CRITICAL CARE AND
SLEEP MEDICINE
UNIVERSITY OF WASHINGTON
SEATTLE, WA 98104

EVANS, CHRISTOPHER M, PHD
ASSOCIATE PROFESSOR
DIVISION OF PULMONARY SCIENCES AND
CRITICAL CARE MEDICINE, SCHOOL OF MEDICINE
UNIVERSITY OF COLORADO DENVER
AURORA, CO 80045

FUNDERBURG, NICHOLAS T, PHD *
ASSISTANT PROFESSOR
DIVISION OF MEDICAL LABORATORY SCIENCE
OHIO STATE UNIVERSITY
COLUMBUS, OH 43210

GOONERATNE, NALAKA S, MD *
ASSOCIATE PROFESSOR
DIVISION OF GERIATRICS
UNIVERSITY OF PENNSYLVANIA
PHILADELPHIA, PA 19104

GUNDRY, REBEKAH L., PHD *
ASSOCIATE PROFESSOR OF BIOCHEMISTRY
DIRECTOR, CENTER FOR BIOMEDICAL MASS
SPECTROMETRY
RESEARCH
MEDICAL COLLEE OF WISCONSIN
MILWAUKEE, WI 53226

HOFMANN BOWMAN, MARION A, MD, PHD
ASSOCIATE PROFESSOR
CARDIOLOGY CLINIC
CARDIOVASCULAR CENTER
UNIVERSITY OF MICHIGAN
ANN ARBOR, MI 48109

KERN, LISA M, MD, MPH
ASSOCIATE PROFESSOR
DEPARTMENT OF MEDICINE
DIVISION OF GENERAL INTERNAL MEDICINE
WEILL CORNELL MEDICINE
NEW YORK , NY 10021

KEY, NIGEL S, MD
PROFESSOR
DEPARTMENT OF MEDICINE
UNIVERSITY OF NORTH CAROLINA AT CHAPEL HILL
CHAPEL HILL, NC 27599

LEDERER, W. JONATHAN, MD, PHD
DIRECTOR AND PROFESSOR
CENTER FOR BIOMEDICAL ENGINEERING
AND TECHNOLOGY
UNIVERSITY OF MARYLAND, BALTIMORE
BALTIMORE, MD 21201

MCGRATH-MORROW, SHARON ANN, MD
PROFESSOR
EUDOWOOD DIVISION OF PEDIATRIC
RESPIRATORY SCIENCES
JOHNS HOPKINS HOSPITAL
THE JOHNS HOPKINS UNIVERSITY
BALTIMORE, MD 21287

MEDOFF, BENJAMIN D, MD
CHIEF, DIVISION OF PULMONARY AND CRITICAL CARE
PULMONARY AND CRITICAL CARE UNIT
MASSACHUSETTS GENERAL HOSPITAL
BOSTON, MA 02114

MYERS, DANIEL DURANT JR., DVM, MPH
DIRECTOR AND ASSOCIATE PROFESSOR
CONRAD JOBST VASCULAR RESEARCH LABORATORIES
SECTION OF VASCULAR SURGERY
DEPARTMENT OF SURGERY
UNIVERSITY OF MICHIGAN
ANN ARBOR, MI 48109

PAKHOMOV, SERGUEI VS, PHD
PROFESSOR
COLLEGE OF PHARMACY
UNIVERSITY OF MINNESOTA
MINNEAPOLIS, MN 55406

PIPINOS, IRAKLIS ILIAS, MD, PHD
PROFESSOR
DEPARTMENT OF SURGERY
UNIVERSITY OF NEBRASKA MEDICAL CENTER
OMAHA, NE 68198

QI, QIBIN, PHD *
ASSISTANT PROFESSOR
ASSOCIATE DIRECTOR, CENTER FOR POPULATION
COHORTS
DEPARTMENT OF EPIDEMIOLOGY AND POPULATION
HEALTH
ALBERT EINSTEIN COLLEGE OF MEDICINE
YESHIVA UNIVERSITY
BRONX, NY 10461

SHEEHAN, JOHN PATRICK JR, MD *
ASSOCIATE PROFESSOR
DEPARTMENT OF MEDICINE
DIVISION OF HEMATOLOGY AND ONCOLOGY
UNIVERSITY OF WISCONSIN SCHOOL OF MEDICINE
MADISON, WI 53706

SROUR, EDWARD F, PHD *
ROBERT J. AND ANNIE S. ROHN PROFESSOR
LEUKEMIA RESEARCH
DEPARTMENT OF MEDICINE
INDIANA UNIVERSITY SCHOOL OF MEDICINE
INDIANAPOLIS, IN 46202

TANABE, PAULA, MPH, PHD, RN
ASSOCIATE DEAN FOR RESEARCH DEVELOPMENT AND
DATA SCIENCE
SCHOOL OF NURSING
DUKE UNIVERSITY
DURHAM, NC 27710

VAZQUEZ PADRON, ROBERTO IRENARDO, PHD
PROFESSOR
DEPARTMENT OF SURGERY
DIVISION OF VASCULAR SURGERY
MILLER SCHOOL OF MEDICINE
UNIVERSITY OF MIAMI
MIAMI, FL 33136

WASSEL, CHRISTINA L, PHD
PRINCIPAL RESEARCH SCIENTIST, APPLIED RESEARCH
PREMIER, INC.
13034 BALLANTYNE CORPORATION PLACE
CHARLOTTE, NC 22877

WAXMAN, JOSHUA, PHD *
ASSOCIATE PROFESSOR
MOLECULAR CARDIOVASCULAR BIOLOGY DIVISION
CINCINNATI CHILDREN'S HOSPITAL MEDICAL CENTER
CINCINNATI, OH 45229

SCIENTIFIC REVIEW OFFICER

MINTZER, KEITH A, PHD
SCIENTIFIC REVIEW OFFICER
OFFICE OF SCIENTIFIC REVIEW/DERA
NATIONAL HEART, LUNG, AND BLOOD INSTITUTE
BETHESDA, MD 20892-7924

EXTRAMURAL SUPPORT ASSISTANT

HALL, JACQUELINE
REVIEW SPECIALIST
NATIONAL HEART, LUNG, AND BLOOD INSTITUTE
NATIONAL INSTITUTES OF HEALTH
BETHESDA, MD 20982

PROGRAM REPRESENTATIVE

AGRESTI, ANTHONY
GRANT MANAGEMENT SPECIALIST
OFFICE OF GRANTS MANAGEMENT
NATIONAL HEART, LUNG, AND BLOOD INSTITUTE
NATIONAL INSTITUTES OF HEALTH
BETHESDA, MD 20892

ARTEAGA, SONIA S, PHD
PROGRAM DIRECTOR
DIVISION OF CARDIOVASCULAR SCIENCES
NATIONAL HEART, LUNG AND BLOOD INSTITUTE
NATIONAL INSTITUTE OF HEALTH
BETHESDA, MD 20892

BARR, ROBIN, DPHIL, PHD
DIRECTOR OF EXTRAMURAL ACTIVITIES
OFFICE OF EXTRAMURAL ACTIVITIES
NATIONAL INSTITUTE ON AGING
NATIONAL INSTITUTE S OF HEALTH
BETHESDA, MD 20814

BROWN, MARISHKA, PHD
HEALTH SCIENTIST ADMINISTRATOR
DIVISION OF LUNG DISEASE
NATIONAL HEART LUNG, AND BLOOD INSTITUTE
BETHESDA, MD 20814

CALER, ELISABET V, PHD
PROGRAM OFFICIAL
DIVISION OF LUNG DISEASES
NATIONAL HEART, LUNG AND BLOOD INSTITUTE
NATIONAL INSTITUTES OF HEALTH
BETHESDA, MD 20892

CAMPO, REBECCA A., PHD
PROGRAM DIRECTOR
DIVISION OF CARDIOVASCULAR SCIENCES
NATIONAL HEART, LUNG AND BLOOD INSTITUTE
BETHESDA, MD 20892

CHANG, HENRY, MD
PROGRAM OFFICER
DIVISION OF BLOOD DISEASES AND RESOURCES
NATIONAL HEART, LUNG, AND BLOOD INSTITUTE
BETHESDA, MD 20892

COOPER, LAWTON S., MD, MPH
HEALTH SCIENTIST ADMINISTRATOR
CLINICAL APPLICATIONS & PREVENTION BRANCH
NATIONAL HEART, LUNG, AND BLOOD INSTITUTE
BETHESDA, MD 29892

DI FRONZO, NANCY L, PHD
PROGRAM DIRECTOR
BLOOD RESOURCES PROGRAM, DBDR
NATIONAL HEART, LUNG AND BLOOD INSTITUTE, NIH
NATIONAL INSTITUTES OF HEALTH
BETHESDA, MD 20892

EINHORN, PAULA T., MD
MEDICAL OFFICE/PROGRAM DIRECTOR
DIVISION OF CARDIOVASCULAR SCIENCES
NATIONAL HEART, LUNG, AND BLOOD INSTITUTE
BETHESDA, MD 20792

HSU, LUCY L.
PROGRAM REPRESENTATIVE
EPIDEMIOLOGY BRANCH
NATIONAL HEART, LUNG AND BLOOD INSTITUTE
NATIONAL INSTITUTES OF HEALTH
BETHESDA, MD 20892

HUANG, LI-SHIN, PHD
HEALTH SCIENTIST ADMINISTRATOR
DIVISION OF CARDIOVASCULAR SCIENCES
NATIONAL HEART, LUNG AND BLOOD INSTITUTE
BETHESDA, MD 20837

KALANTARI, ROYA

KIT, BRIAN K, MD
MEDICAL OFFICER
DIVISION OF CARDIOVASCULAR SCIENCES
NATIONAL HEART LUNG AND BLOOD INSTITUTE
NATIONAL INSTITUTES OF HEALTH
BETHESDA, MD 20817

LAPOSKY, AARON, PHD
PROGRAM DIRECTOR
DIVISION OF LUNG DISEASES
NATIONAL HEART, LUNG, AND BLOOD INSTITUTE
BETHESDA, MD 20892

LIDMAN, KARIN FREDRIKSSON, PHD
PROGRAM OFFICER
OFFICE OF RESEARCH TRAINING AND CAREER
DEVELOPMENT
DIVISION OF CARDIOVASCULAR SCIENCES
NATIONAL HEART, LUNG, AND BLOOD INSTITUTE
BETHESDA, MD 20892

LUDLAM, SHARI, PHD
PROGRAM OFFICER
NATIONAL HEART, LUNG, AND BLOOD INSTITUTE
NATIONAL INSTITUTES OF HEALTH
BETHESDA, MD 20892

NATARAJAN, ARUNA R., MD, PHD
MEDICAL OFFICER
NATIONAL HEART, LUNG, AND BLOOD INSTITUTE, NIH
LUNG BIOLOGY AND DISEASE PROGRAM
BETHESDA, MD 20892-7952

OCHOCINSKA, MARGARET J., PHD
PROGRAM OFFICER
DIVISION OF CARDIOVASCULAR DISEASES
NATIONAL HEART, LUNG, AND BLOOD INSTITUTE
BETHESDA, MD 20892

ONKEN, LISA, PHD
CHIEF
DIVISON OF BEHAVIORAL AND SOCIAL RESEARCH
NATIONAL INSTITUTE ON AGING
NATIONAL INSTITUTE OF FHEALTH, DHHS
BETHESDA, MD 20814

PAPANICOLAOU, GEORGE, PHD
PROGRAM DIRECTOR
DIVISION OF PREVENTION AND POPULATION SCIENCES
NATIONAL HEART, LUNG, & BLOOD INSTITUTE
TWO ROCKLEDGE CENTER
BETHESDA, MD 20892

PRATT, CHARLOTTE, PHD
HEALTH SCIENTIST ADMINISTRATOR
PROGRAM DIRECTOR
DIVISION OF EPIDEMIOLOGY & CLINICAL APPLICATIONS
NATIONAL HEART, LUNG AND BLOOD INSTITUTE
NATIONAL INSTITUTES OF HEALTH
BETHESDA, MD 20892

REDMOND, NICOLE MD
MEDICAL OFFICER
CLINICAL APPLICATIONS AND PREVENTION BRANCH
NATIONAL HEART, LUNG AND BLOOD INSTITUTE
BETHESDA, MD 20817

REINECK, LORA A., MD
MEDICAL OFFICER
DIVISION OF LUNG DISEASES
NATIONAL HEART, LUNG, AND BLOOD INSTITUTE
BETHESDA, MD 20892

REIS, JARED P, PHD
PROGRAM OFFICER
EPIDEMIOLOGY BRANCH
NATIONAL HEART, LUNG, AND BLOOD INSTITUTE
NATIONAL INSTITUTES OF HEALTH
BETHESDA, MD 20892

SARKAR, RITA, PHD
PROGRAM DIRECTOR
DIVISION OF BLOOD DISEASES AND RESOURCES
NATIONAL HEART, LUNG AND BLOOD INSTITUE
BETHESDA, MD 20814

STONEY, CATHERINE, NOT STATED, PHD
PROGRAM OFFICER
DIVISION OF CARDIOVASCULAR SCIENCES
NATIONAL HEART, LUNG, AND BLOOD INSTITUTE
NATIONAL INSTITUTES OF HEALTH
BETHESDA, MD 20892

TIGNO, XENIA, PHD
PROGRAM DIRECTOR
DIVISION OF LUNG DISEASES
NATIONAL HEART, LUNG AND BLOOD INSTITUTE
BETHESDA, MD 20892

VOGT, MATTHEW
EDUCATION SPECIALIST
OFFICE OF EXTRAMURAL RESEARCH POLICY &
OPERATIONS
NATIONAL INSTITUTE OF ALLERGY AND INFECTIOUS DISEA
NATIONAL INSTITUTES OF HEALTH
ROCKVILLE, MD 20852

WANG, WAYNE C., PHD
PROGRAM OFFICIAL
DIVISION OF CARDIOVASCULAR SCIENCES
NATIONAL HEART, LUNG, AND BLOOD INSTITUTE
BETHESDA, MD 20892

WANG, XIBIN, PHD
SCIENTIFIC REVIEW OFFICER
NATIONAL INSTITUTE OF ARTHRITIS,
MUSCULOSKELETAL AND SKIN DISEASES
NATIONAL INSTITUTES OF HEALTH
BETHESDA, MD 20892

WELNIAK, LISBETH A, PHD
PROGRAM OFFICER
DIVISION OF BLOOD DISEASES AND RESOURCES
NATIONAL HEART, LUNG, AND BLOOD INSTITUTE
BETHESDA, MD 20892

WOLZ, MICHAEL, PHD
STATISTICIAN
DIVISION OF CARDIOVASCULAR SCIENCES
NATIONAL HEART, LUNG, AND BLOOD INSTITUTE
BETHESDA, MD 20892

WRIGHT, JACQUELINE, PHD
PROGRAM SPECIALIST
EPIDEMIOLOGY BRANCH
NATIONAL HEART, LUNG AND BLOOD INSTITUTE
NATIONAL INSTITUTES OF HEALTH
BETHESDA, MD 20892

GRANTS MANAGEMENT REPRESENTATIVE

LYONS, MYKLE, MS
GRANT MANAGEMENT SPECIALIST
OFFICE OF GRANTS MANAGEMENT
NATIONAL HEART, LUNG, AND BLOOD INSTITUTE
BETHESDA, MD 20892

* Temporary Member. For grant applications, temporary members may participate in the entire meeting or may review only selected applications as needed.

Consultants are required to absent themselves from the room during the review of any application if their presence would constitute or appear to constitute a conflict of interest.
